# Acute Respiratory Distress Syndrome Requiring Extracorporeal Membrane Oxygenation as the Initial Presentation of Anti-neutrophillic Cytoplasmic Auto-antibody Positive Vasculitis

**DOI:** 10.7759/cureus.6135

**Published:** 2019-11-12

**Authors:** Suhali Kundu, Shaurya Sharma, Ramandeep Minhas, Joshua Scheers-Masters, Paul C Saunders

**Affiliations:** 1 Internal Medicine, Maimonides Medical Center, Brooklyn, USA; 2 Diagnostic Radiology, SUNY Downstate Medical Center, Brooklyn, USA; 3 Rheumatology, Maimonides Medical Center, Brooklyn, USA; 4 Cardiothoracic Surgery, Maimonides Medical Center, Brooklyn, USA

**Keywords:** ards, extracorporeal membrane oxygenation (ecmo), mpo/p-anca, p-anca vasculitis, pauci immune glomerulonephritis, renal biopsy, critical care, cardiothoracic surgery, icu, young

## Abstract

Acute respiratory distress syndrome (ARDS) is a life-threatening inflammatory state of lung injury that can require acute interventions including mechanical ventilation as well as emergent veno-venous extracorporeal membrane oxygenation (VV-ECMO) for management. Etiologies of ARDS are not clearly discernible in certain cases and can vary from sepsis, pneumonia, trauma and intoxication. Anti-nuclear cytoplasmic auto-antibody (ANCA)-associated vasculitis (AAV) is a group of several conditions that can have pulmonary complications including ARDS.

We present a case where the primary manifestation of myeloperoxidase (MPO)-ANCA positive vasculitis was ARDS, in order to highlight the importance of investigating rare vasculitides as the underlying cause of ARDS and the importance of ECMO as an early life-saving intervention for the management of ARDS.

## Introduction

Pulmonary vasculitides are rare heterogenous disease entities characterized by vessel inflammation and destruction. Diagnosis of these disorders is arduous because of their variegated clinical presentation. There are several conditions classified under the general heading of anti-nuclear cytoplasmic autoantibody (ANCA)-associated vasculitis (AAV) including the following: microscopic polyangiitis (MPA), granulomatosis with polyangiitis (GPA) and eosinophilic granulomatosis with polyangiitis (Churg-Strauss syndrome). The incidence of AAV is 15-20 cases per million per year, a prevalence of 90-300 cases per million [1]. Lung lesions are an important feature of AAV. These disease conditions affect multiple organs including the kidneys, lungs, joints, eyes, heart, nervous system and skin [2, 3]. For our interest, this article primarily focuses on the involvement of the lungs. Manifestations differ depending on the specific condition. For example, the hallmark feature for Churg-Strauss is asthma whereas GPA presents with upper and/or lower respiratory tract lesions. MPA most frequently manifests with pulmonary fibrosis and alveolar hemorrhage [2]. Pulmonary involvement is less frequent in MPA than either GPA or Churg-Strauss syndrome. Around 10-30% of patients will develop diffuse alveolar hemorrhage, and although rare, lung involvement in AAV may manifest to acute respiratory distress syndrome (ARDS) [1]. Acute respiratory distress syndrome is a process of non-hydrostatic pulmonary edema with hypoxemia [4]. This paper examines one such case of severe ARDS as the primary manifestation of AAV in a young man.

## Case presentation

**Figure 1:**
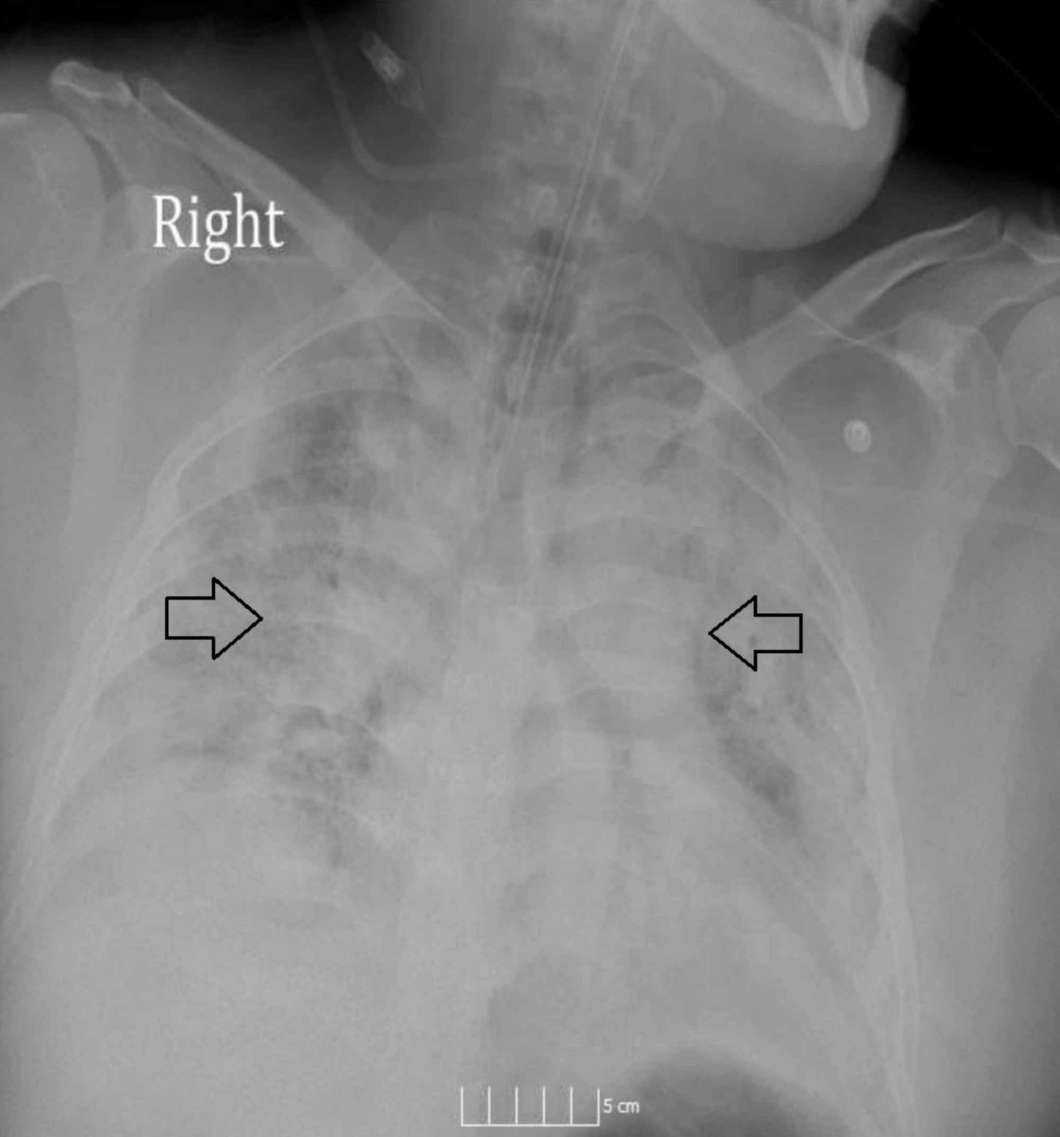
Anteroposterior (AP) chest X-ray showing extensive bilateral airspace disease consistent with acute respiratory distress syndrome (ARDS) on presentation to our hospital.

A 33-year-old man with no significant past medical history presents to the emergency department (ED) with the chief complaint of progressively worsening shortness of breath over 24 hours. He works at a construction site and was not wearing a protective mask while being exposed to cement dust. He was brought in with concerns of inhalation injury. On arrival, he was found to be in severe respiratory distress requiring supplemental oxygen via a nasal cannula, which was escalated to a non-rebreather facemask and further to BiPAP (BiLevel positive airway pressure) due to worsening oxygenation. He remained hypercarbic and hypoxemic on repeat blood gas analysis despite non-invasive ventilation and required intubation for severe hypoxic respiratory failure secondary to ARDS (Figure 1) in the intensive care unit.

Following intubation and mechanical ventilation, the patient remained hypoxemic and hypercarbic requiring emergent veno-venous extracorporeal membrane oxygenation (VV-ECMO). The patient remained on ECMO for seven days, and during this period, the workup for another underlying etiology of ARDS was started as the severity of his symptoms and multiorgan failure could not be explained by the inhalation injury from limited cement exposure.

On admission, the patient was also found to be in acute renal failure with BUN/Cr of 61/2.8 with proteinuria of 30 mg/dl. In addition, the patient had leukocytosis of 20.9 K/UL, anemia with hemoglobin and hematocrit of 6.1 gm/dL and 19.1%, respectively. Further, lactate dehydrogenase (LDH) was elevated to 735 IU/L, with normal haptoglobin of 151 mg/dl and iron studies revealed an iron of 23 mcg/dl (low), ferritin 207.6 ng/ml (high), TIBC 177 mcg/dl (low) and transferrin 126.6 mg/dl (low); transferrin saturation was 12.9% suggestive for anemia of chronic disease/inflammation. Follow-up complete blood count demonstrated an up-trending white blood cell count with increased neutrophils concerning for an infectious process for which broad-spectrum antibiotic coverage was initiated. Concurrent infectious workup did not yield any results and the patient remained febrile while on antibiotics. HIV and hepatitis panels were negative. At this point antibiotics were discontinued and other etiologies of the patient’s persistent fever were explored.

**Figure 2:**
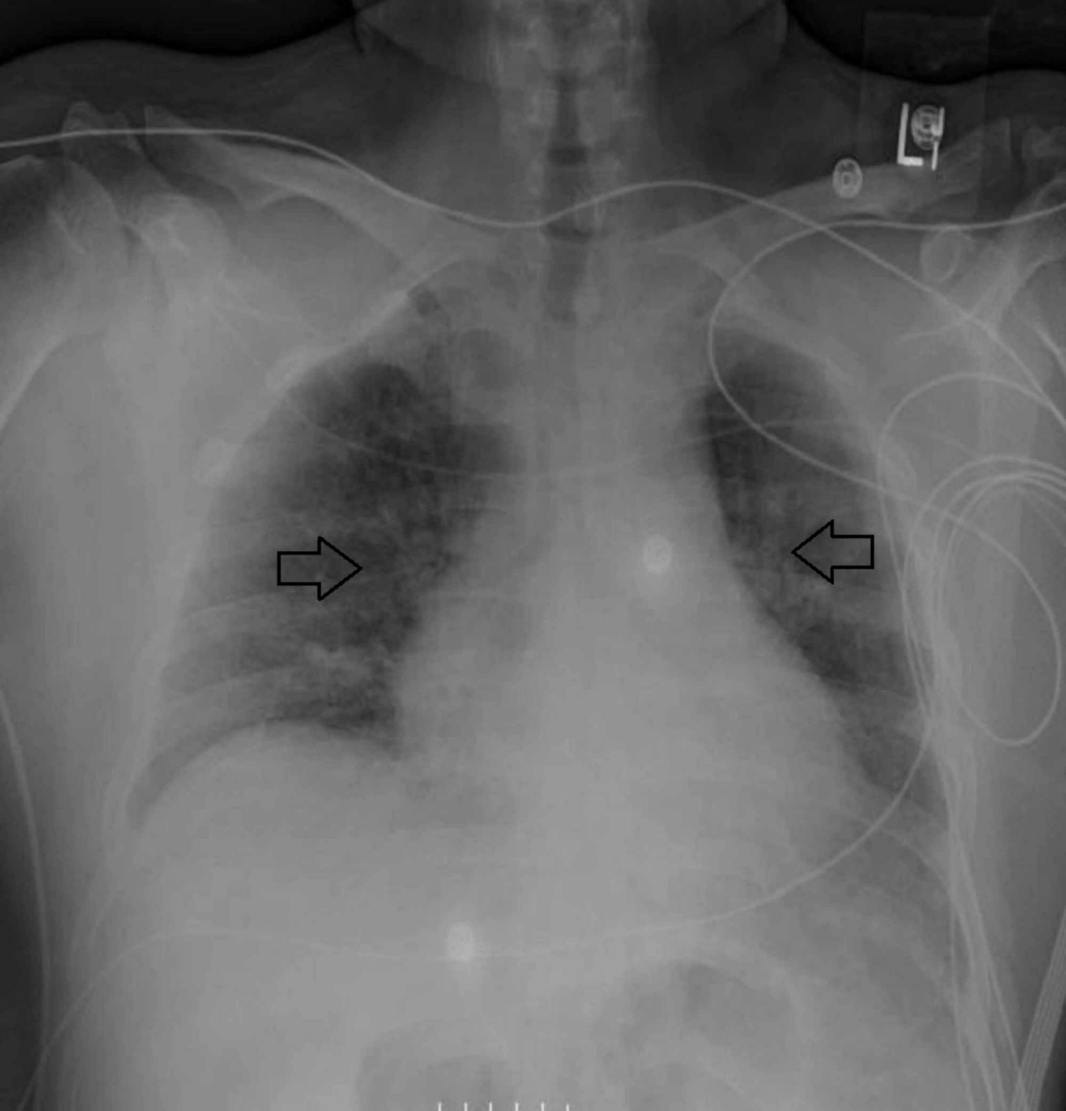
Anteroposterior (AP) chest X-ray showing interval decrease in bilateral lung infiltrates seven days after initiation of extra-corporeal membrane oxygenation (ECMO) and addition of pulse-dose steroids.

Given that the patient was admitted with acute kidney injury and was found to be anemic with evidence of hemolysis, autoimmune etiologies for ARDS were considered and vasculitis serologies were sent; the results of which showed C3 125 mg/dl (normal), C4 25 mg/dl (normal), C-ANCA negative, P-ANCA positive, P-ANCA titer 1:320 and atypical ANCA negative. Given that the patient was P-ANCA positive, Myeloperoxidase (MPO) antibody and Proteinase-3 (PR3) antibody were sent; PR3 Ab was negative while MPO Ab was positive at 117.5 units (high). Glomerular basement membrane antibody was negative. Pulse dose IV steroids were initiated on day seven post admission for the suspicion of ANCA vasculitis which resulted in improvement of airspace disease as seen on follow-up chest X-rays (Figure 2).

**Figure 3:**
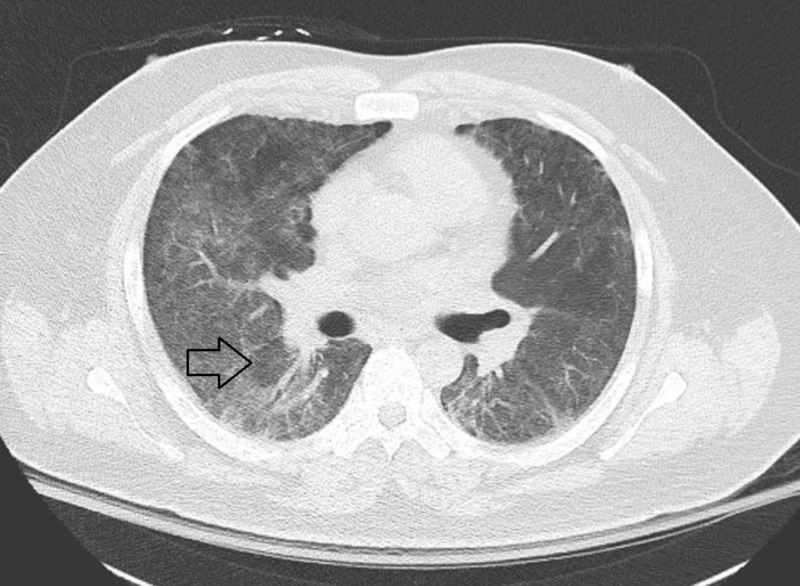
CT chest without contrast: Acute respiratory distress syndrome (ARDS) in ANCA-associated vasculitis (AAV), 10 days post VV-ECMO with radiographic improvement of lung disease and evidence of residual basal interstitial lung disease (ILD). ANCA: Anti-nuclear cytoplasmic auto-antibody; VV-ECMO: Veno-venous extracorporeal membrane oxygenation.

Following three days of IV steroids, oral steroids were continued for the remainder of the hospital course. CT of the chest was repeated at 10 days post VV-ECMO which demonstrated improvement of his extensive lung disease with an evidence of a small amount of residual basilar interstitial lung disease (Figure 3).

**Figure 4:**
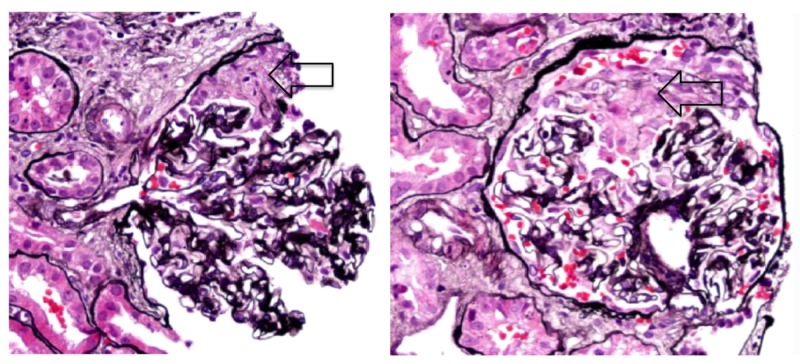
Renal biopsy of our patient with pauci-immune focal necrotizing glomerulonephritis showing crescent formation within glomeruli (arrows). Image courtesy of nephroCORE labs.

Renal biopsy was obtained on day 12 post admission, which revealed pauci-immune focal necrotizing glomerulonephritis, with 22% crescents (Figure 4). The patient was diagnosed with MPO antibody positive ANCA vasculitis with interstitial lung disease (ILD)/Alveolitis and pauci-immune glomerulonephritis. In addition to steroids, we also started Rituximab 12 days post admission following confirmatory pathology results. The patient was discharged 20 days post admission and continues to be on maintenance Rituximab therapy. The patient remained clinically stable and asymptomatic on daily PO Prednisone, and received Rituximab 500 mg intravenously every six months for maintenance therapy. To date, our patient has completed four cycles of Rituximab treatment with improvement of renal function (as depicted by normalization of his serum creatinine and proteinuria). The patient has shown remarkable clinical improvement on follow-up appointments with negligible respiratory symptoms.

## Discussion

Pulmonary involvement in AAV is well documented in the literature but is variegated in its manifestation making it difficult to diagnose and treat. For example, MPA is characterized primarily by ILD, while GPA has pulmonary manifestations that include cavitary masses, nodules, and airway stenosis. Both MPA and GPA may present with alveolar hemorrhage syndrome. The diagnosis of AAV as a cause of lung disease is challenging because of the wide differential diagnosis, including infections, adverse drug injury and idiopathic ILD [5-7]. In a study with 140 patients with AAV who underwent chest CT, Mohammad et al. showed that 80% had pulmonary abnormalities and patients with PR3-ANCA positivity demonstrated central airway disease while MPO-ANCA demonstrated usual interstitial pneumonitis (UIP) patterns. It has also been shown that there is predominance of MPO-ANCA positivity in patients with AAV and ILD [8, 9].

Of the mentioned pulmonary manifestations that occur in AAV, new onset ARDS may be the primary and devastating presentation of undiagnosed AAV. Early intervention for ARDS is lifesaving and for our case immediate ECMO intervention was highly beneficial. ECMO served not only to drastically improve his respiratory function, but also served as a useful tool to stabilize the patient until a definitive diagnosis to the etiology of his ARDS could be established (Table 1).

Loscar et al. highlighted a case in 1997 of GPA in a 19-year-old female whose initial presentation was ARDS and sepsis requiring ECMO intervention [10]. Further studies have shown that routine screening for ANCA antibodies in patients with ARDS can rapidly establish a diagnosis of AAV and early intervention even in patients requiring ECMO is favorable [3, 11].

## Conclusions

The importance of our case lies in the recognition that pulmonary injury, in its most severe form of ARDS, may be the initial presentation of anti-nuclear cytoplasmic auto-antibody (ANCA)-associated vasculitis (AAV). With astute clinical judgement, these rare causes may well be an important differential diagnosis to be considered in patients with ARDS of unknown etiology.

## Appendices

**Table 1:** American-European Consensus Conference (AECC), Berlin and Kigali criteria for acute respiratory distress syndrome (ARDS). Referenced from [12]. PEEP: Positive end-expiratory pressure; PaO_2_: Arterial oxygen tension; FiO_2_: Inspiratory oxygen fraction; SpO_2_: Arterial oxygen saturation measured by pulse oximetry; CT: Computed tomography.

	AECC definition	Berlin criteria	Kigali modification of Berlin criteria
Timing	Acute onset	Within one week of a known clinical insult or new or worsening respiratory symptoms	Within one week of a known clinical insult or new or worsening respiratory symptoms
Oxygenation	PaO_2_/FiO_2_ ≤200 mmHg (dentified as acute lung injury if ≤300 mmHg)	Mild: PaO_2_/FiO_2_ >200 mmHg but ≤300 mmHg; Moderate: PaO_2_/FiO_2_ >100 mmHg but ≤200 mmHg; Severe: PaO_2_/FiO_2_ ≤100 mmHg	SpO_2_/FiO_2_ ≤315
PEEP requirement	None	Minimum 5 cmH_2_O PEEP required by invasive mechanical ventilation (noninvasive acceptable for mild ARDS)	No PEEP requirement, consistent with AECC definition
Chest imaging	Bilateral infiltrates seen on frontal chest radiograph	Bilateral opacities not fully explained by effusions, lobar/lung collapse or nodules by chest radiograph or CT	Bilateral opacities not fully explained by effusions, lobar/lung collapse or nodules by chest radiograph or ultrasound
Origin of oedema	Pulmonary artery wedge pressure <18 mmHg when measured or no evidence of left atrial hypertension	Respiratory failure not fully explained by cardiac failure or fluid overload (need objective assessment, such as echocardiography, to exclude hydrostatic oedema if no risk factor present)	Respiratory failure not fully explained by cardiac failure or fluid overload (need objective assessment, such as echocardiography, to exclude hydrostatic oedema if no risk factor present)
